# Efficacy of Eggshell Membrane in Knee Osteoarthritis: A Systematic Review and Meta-Analysis

**DOI:** 10.3390/nu16162640

**Published:** 2024-08-10

**Authors:** Ana María García-Muñoz, María Salud Abellán-Ruiz, Ana Isabel García-Guillén, Desirée Victoria-Montesinos

**Affiliations:** 1 Faculty of Pharmacy and Nutrition, UCAM Universidad Católica de Murcia, 30107 Murcia, Spain; amgarcia13@ucam.edu (A.M.G.-M.); dvictoria@ucam.edu (D.V.-M.); 2 Faculty of Nursing, UCAM Universidad Católica de Murcia, 30107 Murcia, Spain; msabellan@ucam.edu (M.S.A.-R.); 3 Faculty of Medicine, UCAM Universidad Católica de Murcia, 30107 Murcia, Spain

**Keywords:** eggshell membrane, osteoarthritis, knee pain, nutraceuticals, joint health

## Abstract

Osteoarthritis (OA) is a prevalent, degenerative joint disease, with knee OA being particularly common and impactful. This systematic review and meta-analysis aimed to assess the efficacy of eggshell membrane (ESM) supplementation in improving joint functionality and reducing pain in individuals with knee OA. A comprehensive search was conducted across PubMed, Scopus, Web of Science, and Cochrane Database up to July 2024, following PRISMA guidelines. Seven randomized controlled trials (RCTs) met the inclusion criteria, with five included in the meta-analysis. The studies compared ESM to a placebo, evaluating outcomes based on assessment tools such as the Western Ontario and McMaster Universities Osteoarthritis Index (WOMAC), Visual Analogue Scale (VAS), and Knee Injury and Osteoarthritis Outcome Score (KOOS). Results indicated that ESM significantly reduced pain and improved functionality, with notable improvements in total WOMAC score (effect size −0.34; 95% CI: −0.56 to −0.13; *p* < 0.001) and pain subscale (SMD −0.23; 95% CI: −0.42 to −0.04; *p* < 0.02). The findings support ESM as a promising adjunctive treatment for knee OA, offering a safe, natural supplement to enhance quality of life. Further high-quality RCTs are needed to confirm these results and explore the long-term effects and mechanisms of ESM.

## 1. Introduction

Osteoarthritis (OA), the most common type of arthritis in developed countries, is a chronic and degenerative joint condition of multifactorial etiology, characterized by the gradual loss of articular cartilage, bone remodeling and osteophyte formation [1,2]. It often affects the hands, hips, lower back and feet, but it is in the knees where it is most frequently presented, being one of the most prevalent rheumatic disorders with the greatest impact on the world population [3]. Knee OA is the most common cause of joint pain, limits functional ability, diminishes quality of life and is the main cause of joint replacement surgeries [4].

According to the latest version of The World Health Organization’s Global Burden of Disease Study of 2019, the global burden of OA has increased steadily since 1990, with around 528 million people worldwide living with osteoarthritis [5]. Knee OA especially affects older people, but it can also affect younger people with specific risk factors. Therefore, these data exert significant pressure on society, and an increase is expected in the coming years due to the aging of the population, the increase in obesity and injury rates [5,6].

Conventional treatment of OA includes pharmacological and non-pharmacological interventions, as established by the WHO, based on a multidisciplinary approach primarily focused on pain relief, such as the use of nonsteroidal anti-inflammatory drugs (NSAIDs) [7], weight loss, physical activity, physiotherapy, and in more severe cases, joint replacement surgery to restore movement and improve quality of life, as well as effectively reduce pain [8,9]. However, etiological heterogeneity hinders the development of effective treatment for OA. Even with these recommendations, and due to economic factors, lack of resources, or associated comorbidities, only a percentage of affected individuals experience pain reduction [10].

Currently, alongside traditional treatment, dietary supplements have emerged as a potential adjuvant strategy to counteract pain in chronic disorders such as knee OA or general OA [11]. While guidelines from the National Institute for Health and Care Excellence (NICE) [12], the Osteoarthritis Research Society International (OARSI) [13], and the American College of Rheumatology (ACR) [14] primarily recommend strategies such as physical therapy, weight management, and pharmacological treatments, bioactive compounds under investigation include collagen, glucosamine, and hyaluronic acid or a combination of hyaluronic acid, glucosamine, and chondroitin [15,16]. Interestingly, these compounds are naturally found in the eggshell membrane (ESM), a thin layer located between the calcified shell and the egg white [17].

ESM is mainly composed of fibrous proteins such as collagen types I, V, and X. Additionally, ESM contains bioactive glycosaminoglycans like dermatan sulfate and chondroitin sulfate, hexosamines such as glucosamine, and significant amounts of hyaluronic acid [18]. Typically, a 300–500 mg dose of ESM contains these components, allowing for meaningful comparisons to other preparations already used in patients, such as glucosamine, chondroitin sulfate, hyaluronic acid, and collagen hydrolysates [15,19,20]. Due to its composition, various clinical trials have evaluated ESM as a potential treatment that can promote joint health, reduce pain, and alleviate joint stiffness [19,21,22]. Although these ESM components are found in the normal diet, the concentrated form and higher bioavailability of these compounds in ESM supplements may provide therapeutic benefits that are not achieved through diet alone [23]. Furthermore, ESM contains antioxidant peptides, which have been shown to reduce oxidative stress, thereby potentially enhancing its pain-relieving effects [24]. However, current guidelines from OARSI, ACR, and NICE have not included these compounds in their recommendations for knee OA, highlighting the need for further studies to conclusively determine their potential benefits.

To date, no systematic review and meta-analysis has been conducted to evaluate the effect of ECM in knee OA. Therefore, the aim of this systematic review is to investigate the efficacy of a food supplement extracted from the internal membrane of the eggshell on joint functionality and perceived pain in individuals diagnosed with knee OA.

## 2. Materials and Methods

This systematic review was conducted in accordance with the guidelines set forth by the Preferred Reporting Items for Systematic Reviews and Meta-Analyses (PRISMA). We included Randomized Clinical Trials (RCTs) that evaluated the effectiveness of eggshell membrane in mitigating symptoms associated with knee joint pain. The protocol for this systematic review and meta-analysis was registered with the International Prospective Register of Systematic Reviews (PROSPERO) (registration number: CRD42022365731).

### 2.1. Eligibility Criteria

The selection criteria for this systematic review were established based on the PICOS framework as follows:

P (participants): individuals diagnosed with osteoarthritis and experiencing knee joint pain; I (intervention): administration of eggshell membrane; C (comparison): control groups receiving a placebo or no treatment; O (outcomes): measures related to knee joint pain and functionality; S (study type): randomized controlled trials. The search was limited to articles written in English or Spanish and published in peer-reviewed journals.

Exclusion criteria were as follows:

(a) Studies that combined eggshell membrane with other nutraceuticals;
(b) Studies lacking necessary quantitative data for the meta-analysis, such as means and standard deviations of the measured outcomes;
(c) Studies without a control group;
(d) Studies where patients did not have knee joint pain or were not diagnosed with osteoarthritis;
(e) Non-original articles such as case reports, editorials, opinion pieces, and reviews; and
(f) Duplicate studies, referring to multiple publications reporting on the same study or patient cohort.

### 2.2. Information Sources and Search Strategy

Two independent researchers conducted a systematic search across PubMed, Scopus, Web of Science, and the Cochrane Database, with the search period extending from inception to July 2024. The selection of studies was guided by a structured search strategy that utilized a combination of specific keywords and Boolean operators. These keywords were grouped into two categories, each representing a unique concept: (a) eggshell membrane, and (b) knee joint pain and related conditions. The actual search query was as follows: (“osteoarthritis” OR “knee” OR “knee pain” OR “knee osteoarthritis” OR “joint pain” OR “arthritis” OR “degenerative joint disease”) AND (“egg shell” OR “eggshell” OR “shell membrane” OR “egg shell membrane” OR “eggshell membrane” OR “NEM” OR “natural eggshell membrane” OR “egg supplement”). The search was supplemented by scanning the reference lists of included studies and relevant review articles to identify any additional studies not captured by the database search.

### 2.3. Selection Process

Following the identification of potential studies, we utilized Mendeley (Windows 10 version; Elsevier, Amsterdam, The Netherlands) to eliminate duplicate entries. Two researchers independently conducted the selection process, reviewing each title and abstract to identify potential publications for full-text review. Any disagreements between the two researchers were resolved by a third member of the team.

### 2.4. Data Items and Quality Assessment

A thorough extraction of pertinent variables was undertaken, encompassing factors such as knee pain measures, study design, participant demographics, selection criteria, and specifics of the intervention (i.e., method of administration, dosage, and duration). One researcher (D.V.-M.) performed the extraction, which was then independently verified for accuracy by a second researcher (A.M.G.-M.). In instances of disagreement, a third researcher (M.S.A.-R.) conducted a final review.

The Cochrane risk-of-bias tool (RoB 2.0) was utilized to assess the risk of bias in the included studies [25]. This tool evaluates five domains: (1) bias stemming from the randomization process; (2) deviations from intended interventions; (3) missing outcome data; (4) measurement of the outcome; and (5) selection of the reported result. Two researchers (A.M.G.-M. and A.I.G.-G.) independently assessed the risk of bias, selecting the appropriate RoB 2.0 tool based on the study type (parallel or crossover).

Publication bias was assessed both visually and statistically. A funnel plot was used for visual inspection, aiding in the identification of potential bias in the meta-analysis. Additionally, a more rigorous statistical evaluation was conducted using Egger’s test, with the significance level set at 0.10 [26].

### 2.5. Synthesis Methods

To evaluate the impact of eggshell membrane on knee joint pain, we conducted a series of meta-analyses using either the DerSimonian and Laird method or the inverse of the variance, contingent on the selected methodology (fixed or random effects) [27]. These analyses compared the eggshell membrane treatment with a control group receiving either a placebo, a substance like the eggshell membrane but without the active ingredients, or no treatment at all.

The results were graphically represented using forest plots, along with the corresponding 95% confidence intervals (CIs). For each study, we calculated the standardized mean difference (SMD) and 95% CI, categorizing the SMD as small (0–0.20), medium (>0.20 to 0.50), or large (>0.50).

Pain and functionality measures such as WOMAC, VAS, Numeric Rating Scale for Pain (NRS-P), and KOOS scores were included in the meta-analysis. NRS-P was equated to VAS, and a transformation was applied to KOOS to align it with WOMAC, given that KOOS is scored in the opposite direction (higher scores indicate improvement). Negative values for these measures were considered as indicators of improvement in knee joint pain.

The heterogeneity among the clinical trials included in this meta-analysis was assessed using the *I*^2^ statistic, which was classified as not significant (<40%), moderate (40–60%), substantial (60–75%), or considerable (75–100%) [28]. If *I*^2^ was not statistically significant (*p* > 0.05), the fixed effects model was used for statistical analysis. Conversely, if *I*^2^ was statistically significant, the random effects model was applied.

Standard deviations were derived from standard errors when necessary. This combination of results was performed following the guidelines from the Cochrane handbook [29].

All statistical analyses were performed using Stata (version 16.1; StataCorp, College Station, TX, USA). The level of statistical significance was set at *p* < 0.05.

## 3. Results

### 3.1. Study Selection

**Figure 1:**
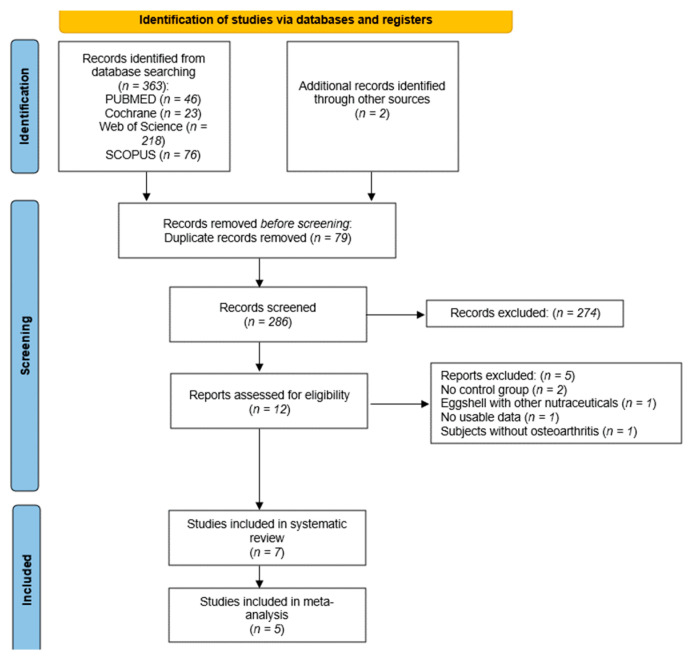
Flow chart.

Database searches yielded a total of 363 records (Figure 1). After removing 79 duplicates, 286 records remained for screening. Upon review of the titles and abstracts, 274 records were excluded as they did not align with the specific objective of this review. This left 12 studies for full-text evaluation, from which 5 were excluded. Two of the studies were excluded because they did not have a control group [30,31], one was excluded due to the combination of eggshell membrane with other nutraceuticals [22], one was excluded due to lack of usable data [32], and another was excluded because the patients did not have osteoarthritis [33]. Ultimately, seven studies were included in the systematic review [15,19,20,21,34,35,36] and five in the meta-analysis [15,19,20,21,34].

### 3.2. Study Characteristics

Table 1 presents the results of the systematic review, which included seven clinical trials investigating the effectiveness of eggshell membrane in treating osteoarthritis. Of these, five studies employed a parallel design [15,19,20,21,36] and two utilized a crossover design [34,35]. The systematic review encompassed studies from four distinct countries. Two studies originated in Spain [19,36], another was conducted in Turkey [34], three were based in the USA [20,21,35], and the final study was carried out in the Netherlands [15].

In terms of participant demographics, the average age across all studies was approximately 53.9 years. Women constituted a mean percentage of 63.8% in the studies. Furthermore, the average Body Mass Index (BMI) of participants, calculated from the available data, was approximately 29.6 kg/m^2^.

The studies included in the review utilized various dosages of eggshell membrane. One study employed both a high dose (HD) of 500 mg and a low dose (LD) of 300 mg [19]. Three studies administered a dosage of 500 mg [21,34,36]. Two other studies used a dosage of 450 mg [20,35]. Lastly, one study used a dosage of 300 mg [15].

The studies also employed a range of outcome measures to assess the effectiveness of eggshell membrane in treating osteoarthritis. The WOMAC was utilized by five studies [19,20,21,34,36]. Two studies incorporated the VAS [19,35]. One study assessed outcomes using the KOOS and NRS-P [15].

In terms of results, all studies reported significant improvements in pain and/or function for participants receiving eggshell membrane compared to those receiving a placebo. Notably, one study reported improvements within 5 days [20], while another observed rapid improvements within just 10 days of supplementation [21]. Improvements within a range of 7 to 30 days were reported in one study [34]. One study demonstrated a significant reduction in pain at the end of the study, with the high-dose group showing a particularly significant reduction compared to the control group [19]. One study reported long-lasting improvements in “Pain” and “Daily Life” functioning, with the pain relief effects peaking after 3 weeks [15]. Another study reported significant improvements in joint function and comfort during daily activities, with improvements in the range of motion observed during the 4-week study period [35]. Finally, Casado-Santos et al. [36] reported significant improvement overall with significant reductions in knee pain and improved physical function observed over the 60-day study period These improvements were maintained or further improved until the end of each study period.

### 3.3. Risk of Bias in Included Studies

**Figure 2:**
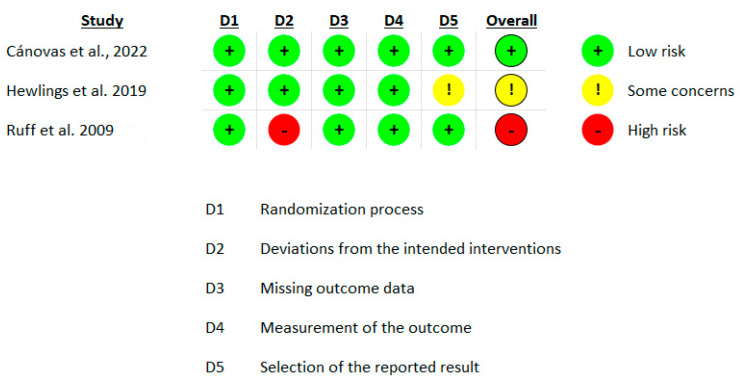
Risk of bias assessment (per protocol analysis, parallel design) using ROB 2.0 [19,20,21]

**Figure 3:**
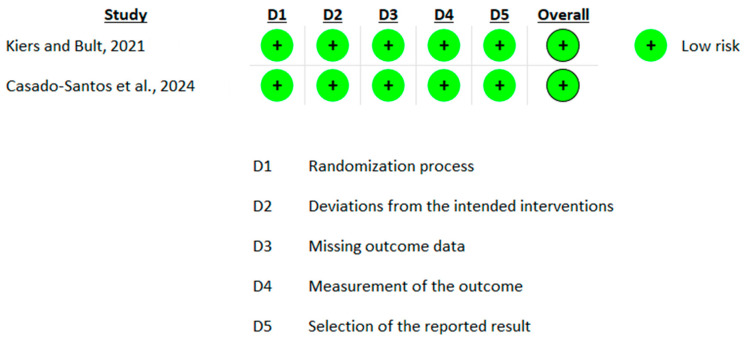
Risk of bias assessment (intention-to-treat analysis, parallel design) using ROB 2.0 [15,36]

**Figure 4:**
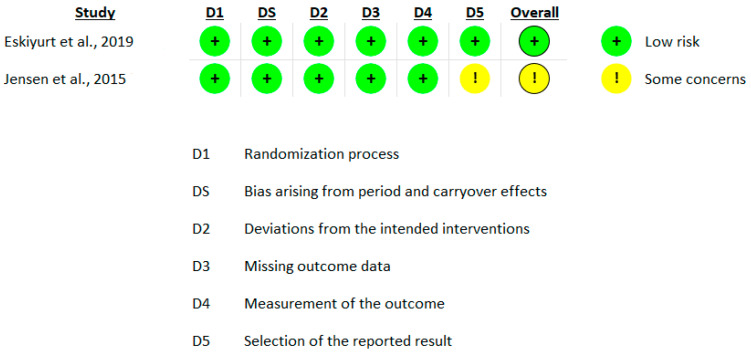
Risk of bias assessment (per protocol analysis, crossover design) using ROB 2.0 [34,35]

The risk of bias assessment for the included studies revealed a varied distribution of bias levels. Four studies demonstrated a low risk of bias [15,19,34,36], indicating that these studies are unlikely to significantly alter the results. These studies adequately addressed all key domains without major concerns. Two studies exhibited a moderate risk [20,35] primarily due to concerns in domain 5, “selection of the reported result”. This indicates that while these studies had some concerns regarding selective reporting, these issues were not sufficient to invalidate the results. One study presented a high risk of bias [21], primarily due to issues in domain 2, “deviations from the intended interventions”, where low adherence to the intervention was observed (Figure 2, Figure 3 and Figure 4).

### 3.4. Effects of the Intervention

The articles incorporated into these meta-analysis included all studies that evaluated the efficacy of eggshell membrane in knee joint osteoarthritis. Five independent meta-analyses were conducted evaluating: the total WOMAC score; subscales of the WOMAC and KOOS tests: the pain measurement subscale, the stiffness subscale and the functional capacity subscale; and pain assessment by VAS.

**Figure 5:**
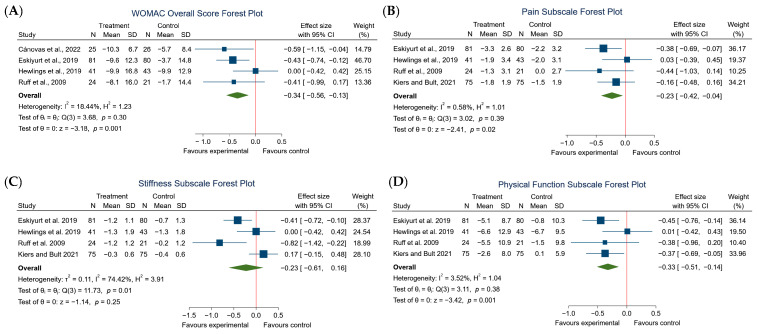
Forest plot comparisons of the effects of eggshell membrane in knee joint osteoarthritis versus placebo on: (**A**) total WOMAC scale score; (**B**) pain subscale; (**C**) stiffness subscale; (**D**) physical function subscale. Square: Represents the result of individual studies (larger squares indicate studies with more weight). Diamond: Summarizes the combined effect of all the studies included in each meta-analysis [15,19,20,21,34].

Improvements in pain and functionality were observed in all studies. The meta-analysis of the total WOMAC score confirmed these results (Figure 5), showing a significant reduction in the total test score (effect size −0.34; 95% CI: −0.56 to −0.13; *p* < 0.001). However, the studies included in this meta-analysis showed a high degree of homogeneity (*I*^2^ = 18.44%).

As shown in Figure 5, four studies were included in the meta-analysis of the pain subscale. The meta-analysis showed a significant reduction in this parameter (SMD −0.23; 95% CI: −0.42 to −0.04; *I*^2^ = 58%; *p* < 0.02).

Pain was also assessed using the VAS. Two trials assessed pain using this tool. The meta-analysis did not show a significant reduction in this variable (SMD −0.92; 95% CI: −3.31 to 1.47; *I*^2^ = 97.51%; *p* = 0.45; Figure S1).

The meta-analysis of the stiffness subscale is shown in Figure 5. No significant reduction was observed (SMD −0.23; 95% CI: −0.61 to 0.16; *I*^2^ = 74.42%; *p* = 0.25).

Finally, the functional capacity subscale showed statistically significant differences with a medium effect size on this score (SMD −0.33; 95% CI: −0.51 to −0.14; *I*^2^ = 3.52%; *p* < 0.001). All articles included in the meta-analysis of the functional ability subscale showed a reduction in the functional ability subscale score, implying an improvement in functional ability (Figure 5).

### 3.5. Publication Bias

Publication bias was visually assessed by funnel plot and confirmed by Egger’s test. The funnel plots for WOMAC Overall Score, Pain subscale and Physical Function subscale showed no evidence visual bias in the funnel plots or Egger’s test bias (*p* > 0.10) (Figures S2–S4 in the Supplementary Materials). In the stiffness subscale, the funnel plot suggests publication bias (Figure S5) although the Egger’s test result showed no statistically significant differences (*p* = 0.249). The pain funnel plot assessed by VAS, risk of bias was observed in both the funnel plot and Egger’s test (*p* = 0.08) (Figure S6).

## 4. Discussion

To the best of our knowledge, this is the first meta-analysis focused on the efficacy of ESM in treating knee OA. From the seven included studies, we extracted data on various pain and functionality measures, with a total of five studies being suitable for meta-analysis. These studies originated from different countries, providing a diverse sample of participants. The findings consistently indicated that ESM significantly reduces knee pain and improves functionality, aligning with current clinical guidelines for managing OA.

The findings of our meta-analysis provide substantial support for the use of ESM in managing knee OA. This aligns well with the current clinical guidelines, particularly those established by ACR, OARSI, and the European League Against Rheumatism (EULAR). These guidelines emphasize the importance of non-pharmacological interventions and dietary supplements as adjunctive therapies for OA. Exercise is universally recommended as a core treatment for OA due to its significant benefits in improving pain and functionality [13,37,38]. In our meta-analysis, ESM showed consistent improvements in pain and functionality measures, such as the WOMAC, VAS, and KOOS [15,19,20,21,34]. For instance, the meta-analysis of the total WOMAC score demonstrated a significant reduction in the total test score with an effect size of −0.34 (95% CI: −0.56 to −0.13; *p* < 0.001), which underscores the potential of ESM to complement physical exercise by providing additional relief from symptoms, especially for patients who may struggle with exercise alone due to severe pain or limited mobility.

The ACR and OARSI guidelines recommend weight loss for overweight patients with knee or hip OA, as excess weight exacerbates joint stress and pain [13,37]. However, achieving significant weight loss through dietary interventions alone can be challenging. Our meta-analysis did not specifically focus on weight loss interventions but highlighted the potential of ESM as a supportive dietary supplement that can alleviate pain. While weight loss remains a crucial component in reducing mechanical joint stress and subsequent pain and inflammation, ESM supplementation offers an additional benefit by providing symptom relief. This aligns with the findings of other meta-analyses, such as that of Christensen et al. [39], which reported modest pain relief from weight loss interventions but noted the difficulty in achieving clinically relevant weight loss. Thus, ESM can be considered a valuable adjunct in comprehensive OA management, complementing the essential recommendation of weight reduction without implying its substitution.

Current guidelines recommend the use of pharmacological treatments such as NSAIDs for short-term pain relief [13,37,38]. However, these medications often come with side effects, especially with long-term use. Our findings indicate that ESM offers a significant reduction in pain and improvement in functionality, with a favorable safety profile. For example, the meta-analysis of the pain subscale showed a significant reduction (SMD −0.23; 95% CI: −0.42 to −0.04; *p* < 0.02), highlighting its potential as a safer alternative or complement to pharmacological treatments for long-term management.

EULAR, ACR, and OARSI guidelines emphasize the importance of self-management and educational interventions to empower patients in managing their condition [13,37,38]. ESM can be integrated into these self-management plans, providing patients with an easily accessible and natural supplement to enhance their quality of life. The significant improvements observed in pain and functionality measures underscore the potential of ESM to support self-management strategies effectively. For example, Jensen et al. [35] reported that participants experienced significant improvements in joint function and comfort during daily activities, with increased physical activity observed during the 4-week study period.

Interestingly, our meta-analysis reveals that ESM, a natural supplement, consistently improves OA symptoms across multiple studies with a low risk of adverse effects. This finding suggests that ESM could be a viable and safe aid for patients seeking natural therapies, complementing the treatments recommended by organizations such as OARSI. For instance, Ruff et al. [33] found that ESM supplementation significantly improved pain and stiffness scores compared to placebo at all time points, with rapid improvements observed within just 10 days of supplementation.

Moreover, the anti-inflammatory properties of ESM could provide an additional mechanism for its effectiveness in reducing OA symptoms. The presence of key bioactive compounds in ESM, such as collagen, elastin, and glycosaminoglycans, supports joint health and may contribute to the observed clinical benefits. These compounds are known to play crucial roles in maintaining the structural integrity and function of joint tissues [36,40,41,42].

### Limitations

Several limitations of this meta-analysis should be acknowledged. The small number of included studies and the variability in sample sizes may affect the robustness and generalizability of the findings. Additionally, the included studies employed different dosages and durations of ESM treatment, which could contribute to heterogeneity in the results. The risk of bias assessment revealed that some studies had a high or moderate risk of bias, primarily due to issues such as deviations from intended interventions and selection of reported results. This highlights the need for more rigorous study designs and comprehensive reporting of results.

Moreover, it is important to note that, although the current manuscript was not supported by grants, all four studies used in the meta-analysis were sponsored by the manufacturers of the eggshell preparations. This sponsorship could introduce potential bias, and therefore, the results should be interpreted with caution. Further independent studies are needed to validate these findings.

We also relied on published data and did not have access to individual patient data (IPD), which would have allowed for more detailed subgroup analyses and a better understanding of the factors influencing the efficacy of ESM. Future meta-analyses should consider incorporating IPD to enhance the precision and applicability of the findings. Additionally, the potential for publication bias must be considered, as studies with positive findings are more likely to be published, which could skew the results.

Furthermore, the authors observed different responses to the therapy in the aspect of the pain subscale. This discrepancy might arise from differences in the focus of the questionnaires used for pain evaluation or potential drawbacks in trial design. This variability underscores the necessity for standardized pain assessment tools and more uniform trial designs to ensure consistency in evaluating the therapeutic effects of ESM.

## 5. Conclusions

In conclusion, this meta-analysis provides strong evidence supporting the efficacy of ESM in reducing knee pain and improving functionality in patients with OA. These findings align with current guidelines emphasizing non-pharmacological interventions and dietary supplements as key components of OA management. However, further high-quality RCTs are needed to confirm these results and address the identified research gaps. ESM represents a potentially valuable addition to the current therapeutic options for OA, offering a safe and effective dietary supplement for managing symptoms.

## Figures and Tables

**Table 1:** Characteristics of included studies on ESM for knee osteoarthritis.

Authors	Country	N	Women *n* (%)	Age (Years)	BMI kg/m^2^	Design RCT	Dosage (mg)	Duration	Disease Severity	Outcome	Results	Conclusions
Cánovas et al. (2022) [19]	Spain	HD = 25 LD = 24 CG = 26	52.0	38.4	25.1	Double blind, parallel	HD = 500 LD = 300	8 weeks	ACR functional grades I-III	WOMAC and VAS	For VAS scale, all groups showed a statistically significant decrease in pain perception at the end of the study. In particular, the high-dose group showed a statistically significant reduction in pain compared to the control group. Regarding the WOMAC results, all groups showed a significant decrease in the WOMAC score, indicating an improvement in functional capacity and quality of life.	Eggshell membrane effectively reduced knee pain and stiffness in osteoarthritis patients, with the response being dose dependent.
Eskiyurt et al. (2019) [34]	Turkey	IG = 81 CG = 80	83.8	57.2	29.5	Double blind, crossover	500	90 days	Kellgren-Lawrence grade 2 and 3	WOMAC	Significant improvements in WOMAC scores (pain, stiffness, function) were observed in the NEM group within 7 to 30 days. The percentage of subjects experiencing greater decreases in WOMAC—pain score was significantly higher in the 90-day NEM group compared to the 60-day group. After 90 days, the original placebo group showed marked clinical improvement upon addition of NEM, resulting in no significant difference in WOMAC scores compared to the original NEM group.	NEM was effective in providing rapid and persistent clinically meaningful improvements in the WOMAC scores for subjects with moderate-to-severe osteoarthritis of the knee. The study also confirmed that NEM was safe and well tolerated, with no occurrence of serious adverse events.
Hewlings et al. (2019) [20]	USA	IG = 41 CG = 43	72.0	53.3	28.2	Double blind, parallel	450	84 days	ACR criteria (minimum 3 criteria)	WOMAC	The change in the composite WOMAC score was statistically different from baseline in the Study Product cohort at all subsequent visits, 3 (day 5), 4 (day 28), 5 (day 57), and 6 (day 86) by *t*-test. The mean change from baseline in the Placebo cohort was not statistically different from baseline in visit 3 (day 5). It was statistically different from baseline in visits 4 (day 28), 5 (day 57), and 6 (day 86) by *t*-test.	The study concluded that the consumption of eggshell membrane showed significant improvement in physical performance, mobility, and joint stiffness within 5 days when compared with a placebo. These improvements were maintained over the 12-week study period. The study also confirmed the safety of product, with no observed human safety concerns.
Ruff et al. (2009) [21]	USA	IG = 29 CG = 31	NR	NR	NR	Double blind, parallel	500	60 days	ACR functional grades I–III	WOMAC	The study found that NEM^®^ supplementation significantly improved pain and stiffness scores compared to placebo at all time points. Rapid improvements were observed within just 10 days of supplementation. While function and overall WOMAC scores also showed improvement, these did not reach statistical significance. The beneficial effects on pain and stiffness were maintained or further improved at the 60-day mark.	The conclusions of the study suggest that eggshell membrane supplementation could be an effective and safe option for the relief of discomfort and inflexibility associated with knee osteoarthritis. The supplement was found to provide rapid relief, with significant improvements observed as early as 10 days after the start of the treatment.
Kiers and Bult (2021) [15]	Netherlands	IG = 75 CG = 75	53.3	63.4	NR	Double blind, parallel	300	84 days	Positive OA diagnosis with self-reported knee pain score ≥ 3 and <9	KOOS and NSR-P	KOOS scores were similar for both the eggshell membrane and placebo groups at the start of the study. However, the effect was significant for two of the five KOOS category scores, namely “Pain” and “Daily Life” functioning, showing long-lasting improvement of 5–8 points on a 0–100 scale of complaint categories. The pain relief effects maximized after 3 weeks and decreased only slightly until measurements finished in week 12. NRS-P scores decreased at a similar rate for both groups during the first six weeks of treatment.	The study concludes that the eggshell membrane extract appears to be effective in alleviating pain and improving daily life functioning in individuals with knee osteoarthritis. The beneficial effects were observed across multiple categories of KOOS scale, indicating a potential for broad impact on quality of life. However, these effects were not mirrored in the results from NRS-P, suggesting that the specific benefits of eggshell membrane may be more nuanced than general pain relief.
Jensen et al. (2015) [35]	USA	IG = 13 CG = 12	63.0	52.5	30.0	Double blind, crossover	450	4 weeks	Mild to moderate physical limitations	VAS	Participants who had been experiencing chronic pain in their knees (among other joints) for at least 6 months reported significant improvements in their condition after consuming water-soluble eggshell membrane. This suggests that water-soluble eggshell membrane may be beneficial in reducing knee pain in individuals with chronic joint conditions.	The consumption of water-soluble egg membrane was associated with significant improvements in joint function, comfort during daily activities, and increased physical activity. Notably, significant improvements in the range of motion were observed for the neck, shoulders, back, hips, knees, and ankles during water-soluble egg membrane consumption compared to placebo.
Casado-Santos et al. (2024) [36]	Spain	IG = 19 CG = 18	56.0	51.4	29.4	Double blind, parallel	300	60 days	Positive OA diagnosis with symptoms of pain, stiffness, or functionality problems	WOMAC	The RM-ANOVA analysis of variance showed a significant overall improvement in patients treated with MKARE^®^, and ESM-RT compared to day 0 in both 30- and 60-days timespans.	MKARE^®^ effectively reduced knee pain and improved physical function in osteoarthritis patients, with significant improvements observed over the 60-day study period.

ACR: American College of Rheumatology; BMI: Body Mass Index; CG: Control Group; IG: Intervention Group; KOOS: Knee Injury and Osteoarthritis Outcome Score; NR: Not Reported; NSR-P: Numeric Pain Rating Scale; OA: osteoarthritis; VAS: Visual Analogue Scale; WOMAC: Western Ontario and McMaster Universities Osteoarthritis Index.

## Supplementary Materials

The following supporting information can be downloaded at: https://www.mdpi.com/article/10.3390/nu16162640/s1. Figure S1, PAIN by EVA Forest Plot; Figure S2, WOMAC Overall Score Funnel Plot; Figure S3, WOMAC Pain Subscale Funnel Plot; Figure S4, WOMAC Physical Function Subscale Funnel Plot; Figure S5, WOMAC Stiffness Subscale Funnel Plot; Figure S6, PAIN by EVA Funnel Plot.
