# Supplementary figures and images for: Efficacy of Eggshell Membrane in Knee Osteoarthritis: A Systematic Review and Meta-Analysis
Source: Nutrients. 2024 Aug 10;16(16):2640. doi: 10.3390/nu16162640 (PMC11356944; doi:10.3390/nu16162640)

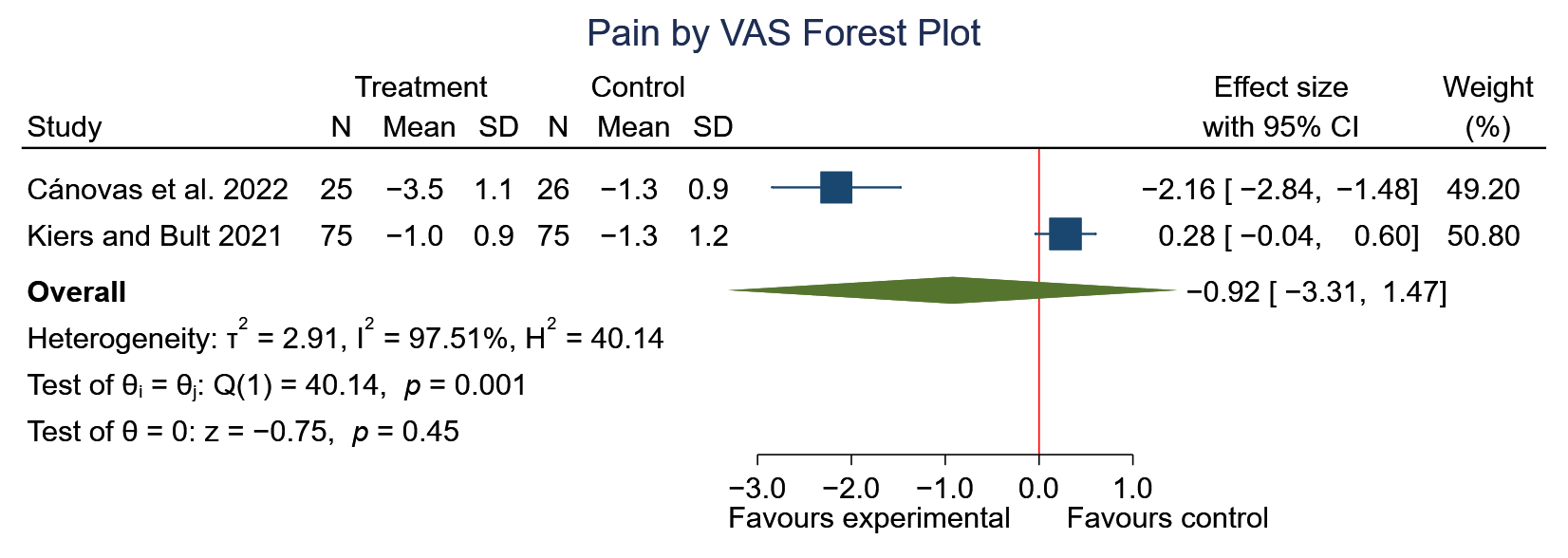

Supplement: Supplementary file 1 [file nutrients-16-02640-s001.zip › Figure S1 - PAIN by EVA Forest Plot.tif]

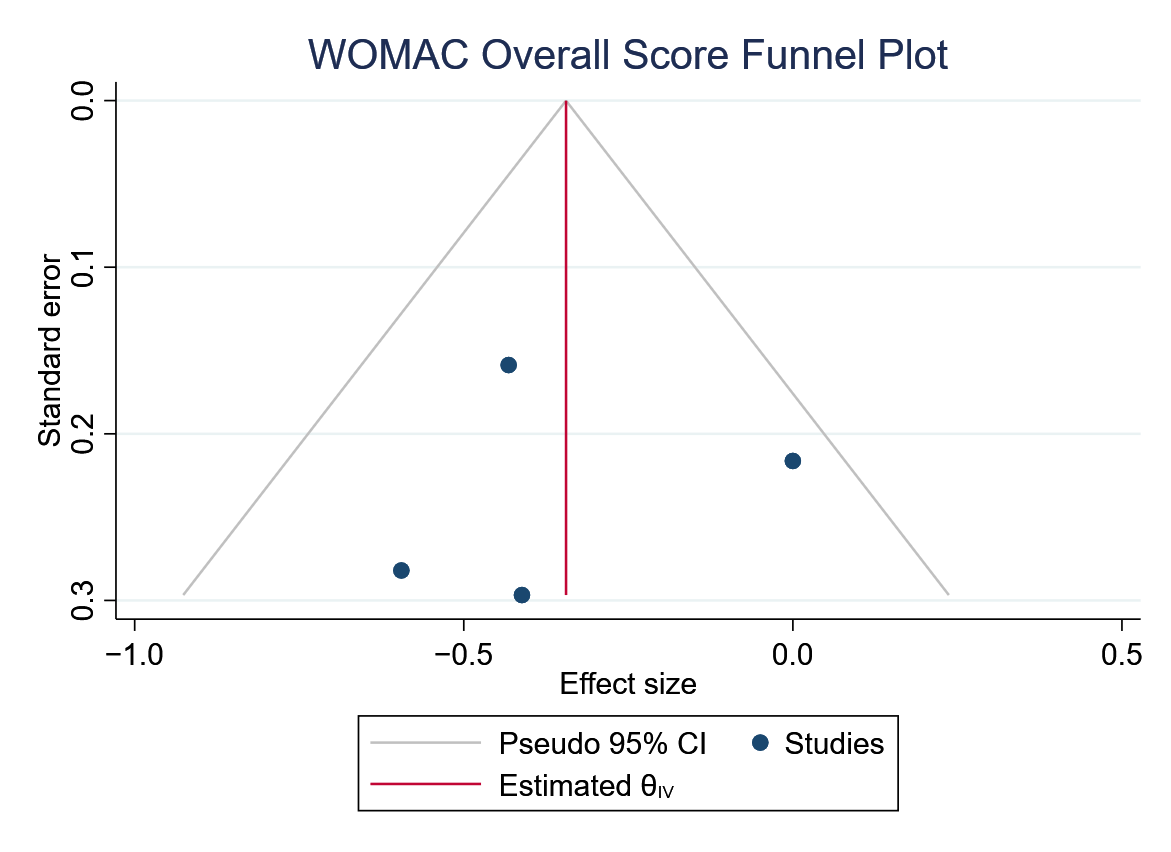

Supplement: Supplementary file 1 [file nutrients-16-02640-s001.zip › Figure S2- WOMAC Overall Score Funnel Plot.tif]

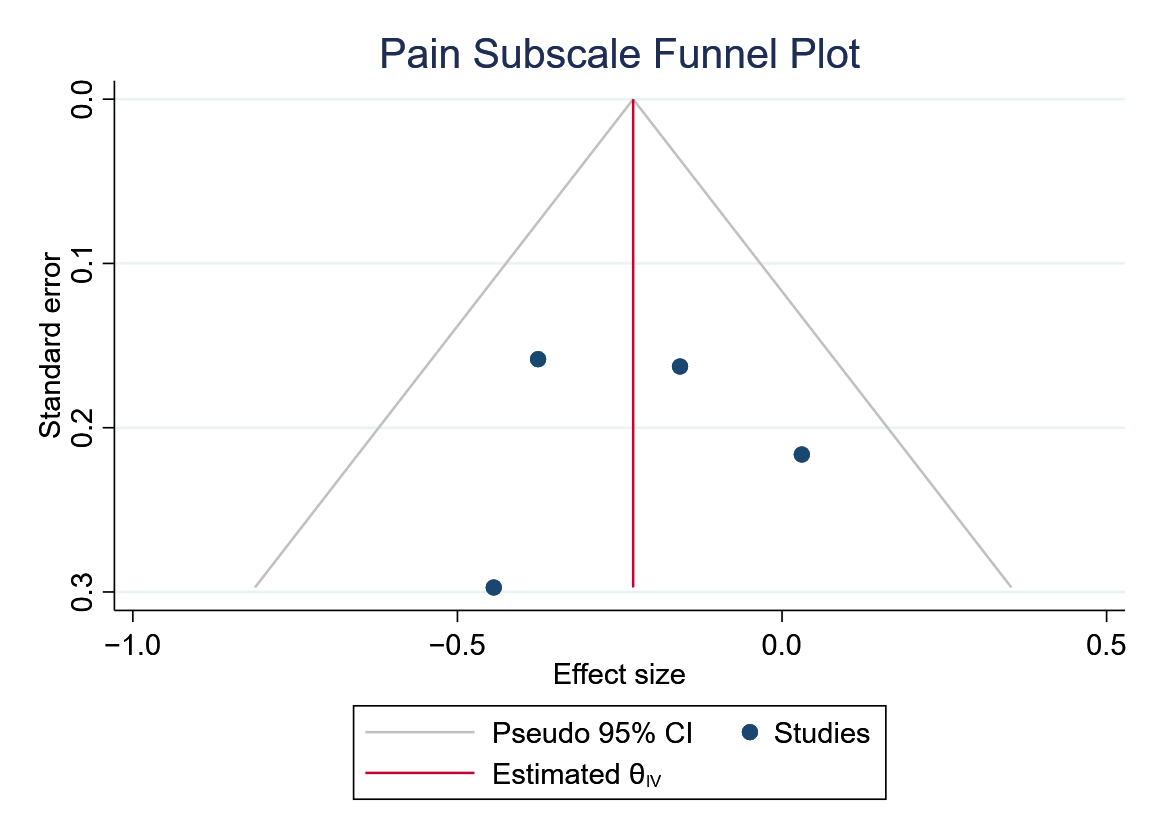

Supplement: Supplementary file 1 [file nutrients-16-02640-s001.zip › Figure S3 - Pain SubscaleFunnel Plot.tif]

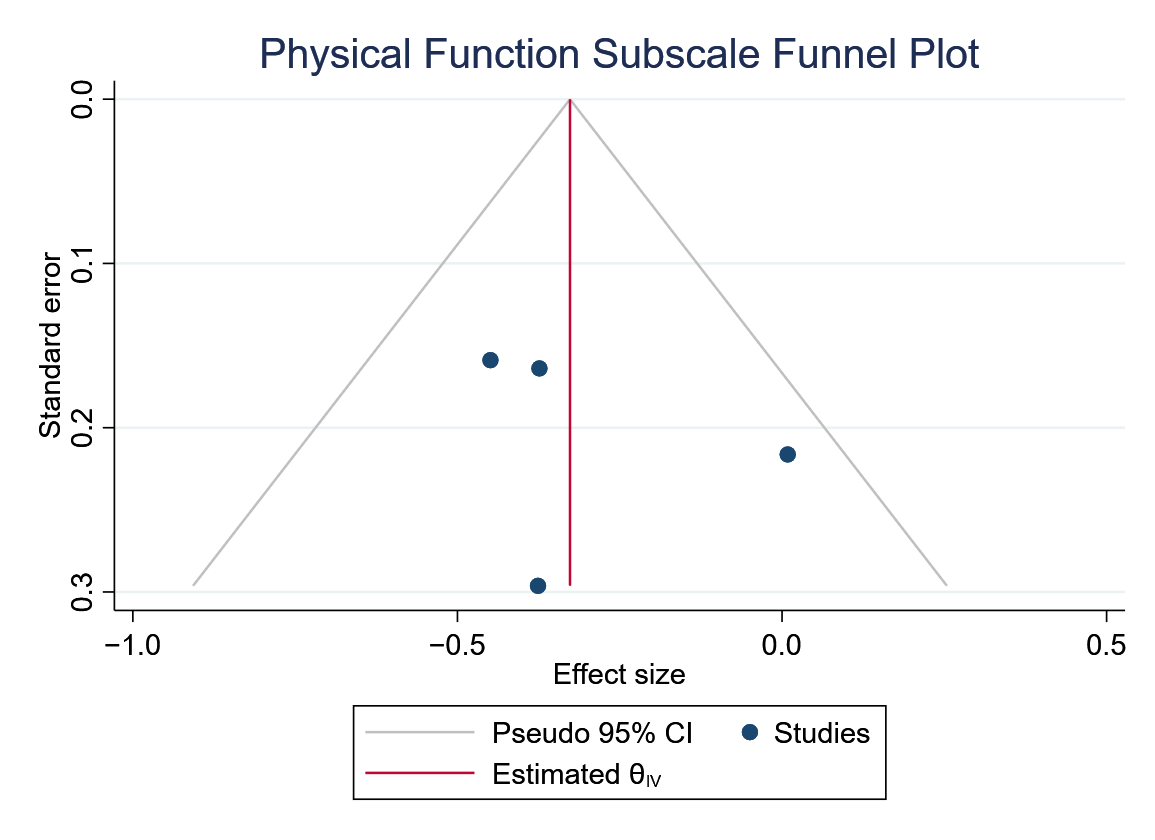

Supplement: Supplementary file 1 [file nutrients-16-02640-s001.zip › Figure S4 -Physical Function Subscale Funnel Plot.tif]

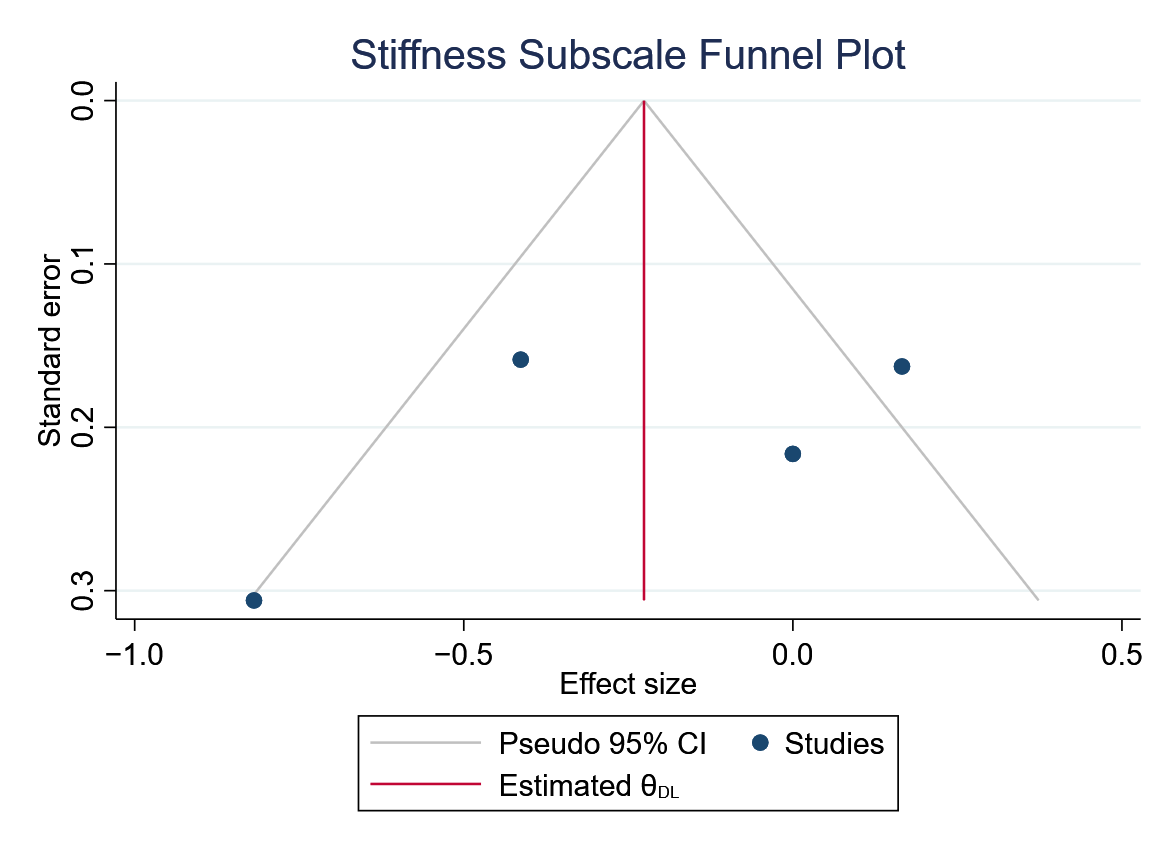

Supplement: Supplementary file 1 [file nutrients-16-02640-s001.zip › Figure S5 -Stiffness Subscale Funnel Plot.tif]

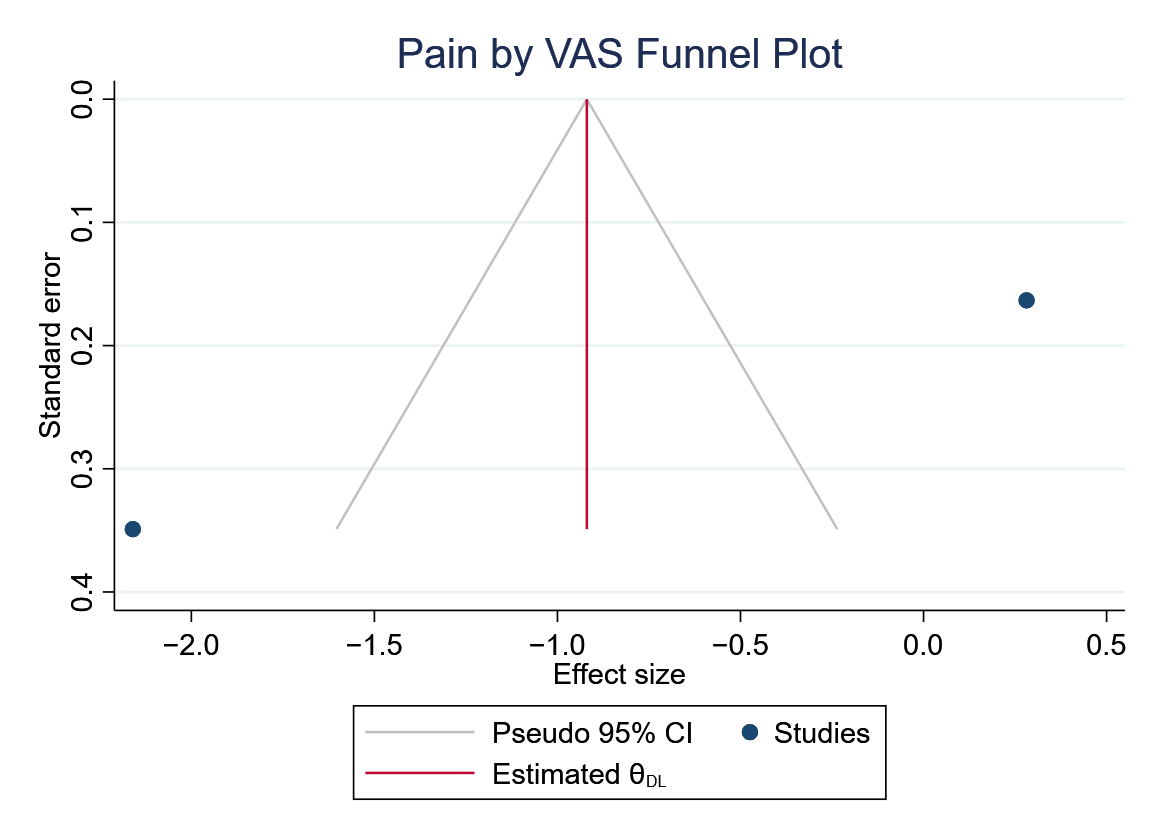

Supplement: Supplementary file 1 [file nutrients-16-02640-s001.zip › Figure S6 - PAIN by EVA Funnel Plot.tif]
